# Implementing active community-based surveillance-response system for Buruli ulcer early case detection and management in Ghana

**DOI:** 10.1371/journal.pntd.0006776

**Published:** 2018-09-12

**Authors:** Collins S. K. Ahorlu, Daniel Okyere, Edwin Ampadu

**Affiliations:** 1 Department of Epidemiology, Noguchi Memorial Institute for Medical Research, College of Health Sciences, University of Ghana, Legon, Ghana; 2 National BU Control Program, Ghana Health Service Korle-Bu, Accra, Ghana; Kwame Nkrumah University of Science and Technology, GHANA

## Abstract

**Background:**

Buruli Ulcer (BU) is one of the most neglected debilitating tropical diseases caused by *Mycobacterium ulcerans*, which causes considerable morbidity and disability. Building on earlier findings that community-based interventions could enhance case detection and reduce treatment dropout and defaulter rates, we established an active surveillance-response system in an endemic sub-district in the Ga West municipality of Ghana to enhance early case detection, diagnosis and treatment to reduce or eliminate severe ulcers and its related disabilities.

**Methods:**

We established surveillance response system, implemented in collaboration with the sub-district disease control officers, selected clinical staff and trained community-based volunteers. The active community-based surveillance- response system was implemented for 12 months. Also, pre and post intervention surveys were conducted to document any change in perceptions on BU in the study population over the period. The baseline and endline surveys were conducted in August 2016 and August 2017 respectively.

**Results:**

On average, each person was seen 11 times in 12 months. In all 75 skin lesions were detected during surveillance rounds, out of which 17 were suspected to be BU and 12 out of the 17 were confirmed as BU using Polymerase chain reaction (PCR). Out of the 12, five, three and four were categories I, II and III lesions respectively. Physical examination was done on 94% of the people seen during the surveillance rounds. Knowledge on BU has also increased in the communities at the end of the study.

**Conclusion:**

The findings from this study have demonstrated that it is possible to establish surveillance-response system for BU and by extension, other neglected tropical diseases to enhance control and elimination efforts through the use of community-based volunteers.

## Author summary

The study revealed that it is feasible to train periphery health workers and community-based volunteers to implement a community-based active surveillance–response system for early buruli ulcer (BU) case detection, diagnosis and treatment at outpatient clinics. At the end of 12 months follow-up, there were 36084 surveillance person contacts made with physical bodily examination done on 93.77% of them. The average surveillance contact per person was 11.3 (91.7%). We believe that this was achieved largely because of the use of community-based volunteers for the surveillance visits. Given the high number of non-BU skin lesions detected during the surveillance period, it is recommended that any BU surveillance-response system must be an integrated one to aid the detection, diagnosis and treatment of other skin conditions to make it more cost effective, this has become even more imperative because the number of BU cases have been declining in most endemic communities in Ghana, since the introduction of antibiotics treatment.

## Introduction

Buruli ulcer (BU) is one of the most neglected debilitating tropical diseases caused by *Mycobacterium ulcerans*. Though, BU-related fatality is very rare, it causes considerable morbidity and disability [1–3], which lead to stigma and anguish among infected individuals and affected families. The disease occurs mainly in remote areas, deprived of basic social infrastructure like health care facilities, potable water and accessible roads [1]. Most endemic countries lack efficient reporting systems, making the assessment of the disease burden unclear [2]. Buruli ulcer has recently received some attention focusing mainly on diagnosis, transmission and clinical management [4–6]. However, there is no active community-based surveillance-response system to generate the essential data necessary to capture and treat cases early enough to prevent complications that contribute so much to morbidity and disability, and cost of treatment to both the health system and affected families. In order to institute an effective public health response to the problem of BU, a strong surveillance system is needed to systematically collect, analyse and interpret data on the disease [7–9].

The epidemiology of BU in endemic countries is not entirely known, due to several factors including the focal distribution of cases, late reporting of cases and lack of health facilities among others in endemic countries of Africa [10]. In Ghana, the first passive surveillance system reported about 1,200 BU cases between 1993 and 1998 and more than 9,000 BU cases were also reported between 2004 and 2014 [9, 11]. A nation-wide active case search that was conducted in 1999 found BU in all the 10 administrative regions of Ghana with an overall prevalence of 20.7 per 100,000 of the population [9]. Currently, BU control in Ghana is mainly through early case detection [9, 12] and clinical diagnosis at peripheral health facilities designated by the National Buruli Ulcer Control Program (NBUCP) followed by laboratory confirmation and subsequent antimycobacterial therapy.

The mode of transmission of the pathogen is still elusive and therefore control relies mainly on case detection and treatment with Streptomycin and Rifampicin for eight weeks, followed by surgery to speed healing and correct deformities [11]. The success of this treatment modality depends very much on detecting suspected cases early for diagnosis and treatment at health facilities [12–14]. However due to socio-cultural and economic factors most cases are detected very late with large wounds with massive cell destruction by the cytopathic toxin, mycolactone [11, 15].

It is known that BU patients in West Africa do not typically seek care from health facilities or do so late with severe ulcers coupled with disabilities leading to under reporting of the disease burden at health facilities [5, 14–17]. However, it has been reported that community-based interventions could enhance case detection and reduced treatment dropout and defaulter rates [17, 18].

Huge resources are being invested to develop new diagnostic tools for BU and to improve on its clinical management. However the discovery of new diagnostic tools and improved clinical management techniques could only be useful when suspected patients seek health care for their conditions, especially at the early stages of the disease, before debilitating complications set in. When identified early, BU can be treated with a high degree of success with rifampicin and streptomycin. This antibiotic combination treatment is noted to have a positive impact on treatment outcomes as it has the potential to cure small lesions and limit surgery for larger lesions [19–21].

The overall aim of this study was to test the feasibility of training periphery health workers and community-based volunteers to implement community-based active surveillance–response system for early BU case detection, diagnosis and treatment on outpatient basis in an endemic sub-district in Ghana.

## Methods

### Ethics statement

The study was reviewed and approved by the Institutional Review Board (IRB) of the Noguchi Memorial Institute for Medical Research (NMIMR) with Federal Wide Assurance Registration FWA 00001824. The protocol was assigned certified protocol number (CPN) 075/14-15. Written informed consent was taken from all participants (Informed consent from all adults aged 18 years and above, Parental consent for all children under 18 years old and Child assent from all children aged between 12 and 17 years.)

### Study design

This was an epidemiologic study designed to test and evaluate a community surveillance-response system in the study area for 12 months. It was a longitudinal study with baseline and endline surveys to compare pre- and post- implementation perceptions among the population.

### Study site

The Ga West Municipality with Amasaman as its capital has an estimated population size of about 215,824 with a growth rate of 3.4 percent [22]. The major economic activity in the municipality is farming, employing about 70% of the people. Other economic activities include fishing, stone quarrying and petty trading.

The district has the highest number of reported BU cases in the Greater Accra Region and is the fifth most BU endemic in the country in 2002. About 1000 cases of BU are reported in Ghana yearly, giving a nationwide prevalence of 20.7/100,000, in 1998. However, the district level prevalence of the Ga South district, where this study took place, was 87.7/100,000 population [23, 24].

The District Hospital at Amasaman has a specialized unit for BU treatment but due to poor road networks and socio-cultural factors, most cases report at the health facility late. The Amasaman hospital continues to be the main health services provider in the district with a few health posts, private clinics, and family planning and maternity homes doted within the municipality. Health care to the rural communities is mostly provided by the Ghana Health Service through monthly outreach services. Among the top five common diseases prevalent in the District are malaria, skin diseases, diarrhoea, HIV/AIDS (Human immunodeficiency virus/ Acquired immunodeficiency syndrome) and BU.

Ten communities namely; Kojo-Ashong, Onyansana, Otuaplam, Yahoman, Okushibiade, Adeyman, Kramo, Domsampaman, Kwashikuma, Odumtia/Akwakyere were identified to participate in the study. These communities were selected with the support of the district health management team and the National BU control programme. The Ga West municipality was selected because it has not only the highest number of reported BU cases in the Greater Accra Region and the fifth most BU endemic in the country but also continue to report the worst forms of the disease, category three ulcers in Ghana. The 10 communities selected were identified by the municipal health directorate as the most endemic communities in the district. Census was conducted in each of the selected communities to register everybody, which then formed the target populations for the establishment of the community-based surveillance -response system.

The current BU control in Ghana is based on the WHO recommended first line treatment for BU using oral rifampicin (10 mg/kg) plus intramuscular streptomycin injection (15 mg/kg), both given daily for 8 weeks under supervision coupled with passive surveillance. Case finding is based on passive surveillance coupled with occasional case search in communities with no on-going active surveillance in endemic communities for early case identification, though early treatment is being promoted.

### Implementation of the surveillance-response system

#### Training of volunteers and health workers

Twenty community based volunteers (trained by the Ghana health service as community-based volunteers), two disease control officers (trained, certified and employed by the Ghana health service as disease control officers) and six selected clinical staff (trained nurses and medical officers who are either working with a health facility or the district health administration) were trained by experienced BU clinicians, including the BU control programme manager and technicians from the Ghana Health Service. They were taught skills for recognizing early suspected BU cases. Additionally, the clinical staffs were also trained to take clinical samples, using Fine Needle Aspiration (FNA) and Swab techniques for laboratory confirmation. However, the samples were actually taken by the decentralized health workers in the study area for laboratory confirmation. Since the volunteers lack confidence to perform FNA and swab, they were exempted from this component of the training but were available as observers. The disease control officers were also trained to be the bridge between the community-based volunteers and the clinic to facilitate quick response to all suspected cases identified through the community-based surveillance-response system for quick diagnosis and treatment.

Six clinical staffs from the three decentralized health facilities in the sub-district were retrained on clinical sample taking for laboratory confirmation as well as on how to manage BU cases on an outpatient basis. They were also equipped to manage cases that were not confirmed as BU appropriately or refer them to the nearest hospital for further examination and treatment. They were also trained on how to generate and manage routine data for decision making to influence their practices. Additionally, the volunteers were trained to perform physical examinations during the surveillance rounds. They were encouraged to talk about BU as a debilitating disease, which is prevalence in the community, and access to effective early treatment at the health facility. It was also emphasized that all suspected cases will be supported to receive appropriate diagnosis and treatment from the nearest health facility.

#### Surveillance-response system

The surveillance-response system was established and implemented from August 2016 to July 2017. Thus 12 monthly rounds, led by community-based volunteers in collaboration with local health workers, were completed in all the selected communities.

#### Baseline and endline surveys

The baseline and endline surveys were conducted in August 2016 and August 2017 respectively, using a pretested structured interviewer administered questionnaire. Respondents were preselected from the various community registers and then visited at home to administer the questionnaire. Six hundred (at baseline) and 526 (at endline) respondents, a minimum of 57 and 52 respondents per community at baseline and endline respectively were interviewed to generate data for comparing baseline responses to the endline responses of community members. These allowed us to determine the effectiveness of the health communication that accompanied the surveillance visits.

### Statistical methods and sample size calculation

At baseline, with an estimated population of 4,000 in the 10 selected communities and assuming that 50% of the people will be willing to participate in the interviews and 5% confidence limits and the design effect of 1.5, we arrived at a sample size of 570 (57/cluster) However, we sampled 60 respondents from each of the 10 communities (600 participants). At the endline survey, a known population size of 3255 was used for the calculation giving a sample size of 52/cluster, thus a total of 520 respondents in the 10 communities, however 526 respondents were interviewed. Participants were randomly selected from the adult population, using a community register, proportionally to represent both sexes.

Data entry and analyses were done using EpiInfo version 7. Findings were presented in descriptive statistics, especially frequencies and percentages. We compare proportions (percentages) from baseline and endline to determine any difference between the two data points. A difference of less than 0.05 (P<0.05) was considered statistically significant.

## Results

A total population of 3070 in 837 households in 10 communities was registered during the census. However, by the end of the 12 month, the population has increased by 185 (6.0%) to 3255. Beside the natural population growth, we believe that the increased in population was due mainly to people moving to settle in the area, which is not far from Accra the capital city of Ghana. Majority of the study participants were females (52.8%). At the end of 12 months, there were 36084 surveillance person contacts made with physical bodily examination done on 33835 (93.8%). The average surveillance contact per person was 11.1 (92.3%) out of the maximum number of 12 expected (Table 1).

**Table 1:** Populations size of selected communities and surveillance contact*.

Name of community	Population size		Surveillance contact	Average person contact in 12 months
	Baseline	Endline		
Adeyman	399	421	4692	11.1
Dom Sampaman	388	399	4152	10.4
Kojo Ashong	404	416	4860	11.7
Kramo	185	198	2208	11.1
Kwashikuma	276	329	3912	11.9
Oduntia	338	352	3996	11.3
Okushibiade	387	401	4464	11.1
Onyansana	227	242	2304	9.5
Otuaplam	264	281	3096	11.0
Yahoman	202	216	2400	11.1
**TOTAL**	**3070**	**3255**	**36084**	**11.1**

* Sorted in column1 in alphabetical order.

Bodily examination was not done on 2249 (2249/36084 x 100 = 6.0%), mainly because participant were; not at home or travelled (70.7%), moved out of the community permanently (19.4%), refused to be examined 136 (6.0%) and others like infants or sick persons made up of only 3.9%. During the 12 months follow up period, a total of 75 skin lesions were encountered, out of which 17 (22.7%) were suspected to be BU by the volunteers. Out of the 17 suspected cases, 12 (70.6%) were confirmed as BU using PCR, making the clinical judgment of the volunteers very good. Out of the 12 confirmed cases, five, three and four were categories I, II and III respectively (Table 2). Thus, two out of three cases were picked from the community early and these could be easily managed at the peripheral health facilities at a lower cost to both the individuals and the health system. All but one (three out of four) of the category III lesions were invited by their relatives who were participating in the study to migrate into the study area in 2017 so that they could benefit from the project activities, especially facilitating laboratory diagnosis and treatment at the nearest health facility. The remaining category III case was hidden in the community for a long time. She reportedly went to the clinic once, many years ago, but had decided against biomedical treatment, and instead resorted to herbal or traditional treatments at home, however, through the surveillance response system, she was rediscovered, diagnosed and helped to receive treatment on an outpatient basis, and her wound has healed. The left leg (75.0%) was the dominant site of confirmed BU lesion (9 out of 12) with one each on the right leg, left and right arms respectively.

**Table 2:** Monthly reported skin lesions, suspected BU and Confirmed BU cases.

Period	Skin Lesions Reported (n = 75)	Suspected BU (n = 17)	Confirmed BU (n = 12)	Category		
				I (n = 5)	II (n = 3)	III (n = 4)
**2016**						
August	24	3	1	1	0	0
September	5	0	0	0	0	0
October	8	3	3	0	2	1
November	15	1	1	1	0	0
December	9	0	0	0	0	0
**2017**						
January	3	3	3	3	0	0
February	7	5	3	0	1	2
March	0	0	0	0	0	0
April	0	0	0	0	0	0
May	0	0	0	0	0	0
June	4	2	1	0	0	1
July	0	0	0	0	0	0

Majority (62.5% at baseline and 64.5% at endline) of the respondents had primary level education. As expected, majority (71.3% at baseline and 61.6% at endline) of the respondents belong to the Ga speaking ethnic group. The Christian religion was the most professed religion reported among respondents; 81.2% at baseline and 84.6 at endline (Table 3). In line with the dominant ethnic groups in the area, majority of the respondents (72.8 at baseline and 66.2% at endline) referred to the disease in the Ga/Agangbe local language as *Odonti hela/ Aboagbonyo*. Others include *Detsifudor/detsifubi* in Ewe language (12.4% at baseline and 14.3% at endline) and *Kisi kuro/Asawa kur*o in Akan language (2.5% at baseline and 3.2% at endline). Also, 11.2% at baseline and 15.0% at endline referred to it as Buruli. It is worth reporting that all the dominant local names could be translated literally to mean cotton wool wound or bad wound.

**Table 3:** Socio-demographic characteristics of respondents at baseline and endline*.

Variable	Baseline	End-line	P-Value
	Frequency (%) n = 600	Frequency (%) n = 526	
**Education**			
No education	156 (26.0)	99 (18.8)	0.005
Others (Adult literacy)	6 (1.0)	2 (0.4)	0.378
Post-Secondary	23 (3.8)	11 (2.1)	0.126
Primary/JSS	375 (62.5)	339 (64.5)	0.538
Secondary/SHS	40 (6.7)	75 (14.3)	<0.001
**Ethnicity**			
Akan	33 (5.5)	68 (12.9)	<0.001
Ewe	128 (21.3)	110 (20.9)	0.921
Ga/Adangbe	428 (71.3)	324 (61.6)	<0.001
Others	11 (1.8)	24 (4.6)	0.014
**Occupation**			
Farming	127 (21.20)	109 (20.7)	0.901
Employed in the formal sector (e.g. Teachers)	9 (1.5)	3 (0.6)	0.220
Others (motor rider etc.)	6 (1.0)	81 (15.4)	<0.001
Petty Trading	192 (32.1)	201 (38.2)	0.035
Sand winning/casual labourer	42 (7.0)	47 (8.9)	0.277
Unemployed	70 (11.7)	85 (16.2)	0.037
**Religion**			
Christianity	487 (81.2)	445 (84.6)	0.149
Islamic	72 (12.0)	60 (11.4)	0.830
No Religion	30 (5.0)	11 (2.1)	0.015
Traditional	11 (1.8)	10 (1.9)	0.893
**Sex**			
Female	315 (52.50)	263 (50)	0.436
Male	285 (47.50)	263 (50)	0.436
Mean Age (±SD)	43.4 (±13.9)	38.0 (±12.5)	0.075

* Sorted in column1 in alphabetical order

Majority of the respondents (89.8% at baseline and 92.6% at endline) said they know about Buruli ulcer as a disease that affects people in the community and have local names for it. The dominant local names reported—*adonti hela* (Ga), *detsifu dor* (Ewe) and *asawa kro* (Twi), could be translated literally to mean cotton wool wound and this could be linked to the cotton wool-like necrotic tissues that are usually found around the edges of BU wounds. The BU related knowledge was acquired from varied sources. The most commonly reported source of knowledge was to *know someone with the infection*, 71.2% at baseline and 53.6% at endline (Table 4).

**Table 4:** Source of BU related Knowledge*†.

Sources	Baseline	Endline	P-Value
	Freq. (%) n = 539	Freq. (%) n = 487	
Community member talk about it at our meetings	66 (12.2)	128 (26.3)	<0.001
Hospital/clinic workers	9 (1.7)	43 (8.8)	<0.001
Know someone who has BU	384 (71.2.)	261 (53.6)	<0.001
Radio/TV/news papers	30 (5.6)	28 (5.8)	0.991
Suffered from it	83 (15.4)	38 (7.8)	<0.001

* Sorted on column1 in ascending order

† Multiple choices were allowed.

Majority (69. 8% at baseline and 73.4% at endline) of the respondents said they could recognize BU at an early stage before it becomes a wound/sore or ulcerated. Among those who said they could recognize early BU, *Nodule* (87.1% at baseline and 96.9% at endline) was the most commonly reported sign. This was followed by *Itching* (10.3% at baseline and 9.8% at endline). Others were; *Papule* (1.9% at baseline and 1.3% at endline), *Plaque* (1.7% at baseline and 2.9% at endline) and *Oedema* (1.2% at baseline and 1.0% at endline).

Various treatment options were reported; chiefly among them was Hospital/clinic treatment (78.3% at baseline and 72.4% at endline). Others were; Traditionalists/Herbalists (34.4 at baseline and 22.2% at endline), Home prepared herbal medicine (17.5% at baseline and 5.7% at endline, self-medication with biomedicine (3.0% at baseline and 1.7% at endline with faith healers reported by two people only at baseline.

Majority (86.2% at baseline and 74.1% at endline) mentioned that BU can be prevented. Prominent prevention methods mentioned included; Cleaning the Environment (30.6% at baseline and 50.8% at end-line), Avoid bathing in the river (32.5% at baseline and 36.9% at end-line) and Regular use of biomedicine; 38.3% at baseline and 27.7% (Table 5).

**Table 5:** BU prevention means reported by respondents*†.

Preventive Means	Baseline	Endline	P-Value
	Freq. (%) n = 517	Freq. (%) n = 390	
Avoid bathing in the river	168 (32.5)	144 (36.9)	0.187
Cleaning the Environment	158 (30.6)	198 (50.8)	<0.001
Eating good food	56 (10.8)	31 (8.0)	0.179
Getting spiritual protection	7 (1.4)	0 (0.0)	0.055
Not offending evil persons	24 (4.6)	0 (0.0)	<0.001
Others	6 (1.2)	2 (0.5)	0.497
Regular use of biomedicine	198 (38.3)	108 (27.7)	0.001
Regular use of herbal medications	70 (13.5)	26 (6.7)	0.001
Stop sand wining in the area	51 (9.9)	24 (6.2)	0.059
Vaccination	66 (12.8)	35 (9.0)	0.090

* Sorted on column1 in ascending order

† Multiple choices were allowed

At baseline, majority (62.8%) of the respondents said they were not aware of any active BU control activity in their communities. However, this had changed at the endline, where majority (55.1%) reported spontaneously that they were aware of a control activity going on in the study communities. When respondents were asked to explain the control activities in their own words, the dominant statement is represented in the following narrative “I know someone who moves from house to house to examine people for the disease and then refer those with the disease to the clinic for treatment.” This statement indicates that people were aware of the surveillance-response system that was implemented in these communities.

As expected, malaria was reported by the majority (88.5% at baseline and 86.88% at endline) as the single most common disease in all the study communities. This was not surprising as the area is noted for high malaria prevalence in the Greater Accra region, where malaria is generally low due to its urban nature. Though, only few respondents mentioned Buruli ulcer as a common disease (6.8% at baseline and 3.8 at end-line), it is important to know that it was considered as an important health problem in the study area. Other health problems mentioned at both baseline and endline were waist pains, stomachache, headache, eye problem and hypertension.

All respondents at endline testified that volunteers come to their homes on monthly basis. However, 92.01% reported that the volunteers did talk to them about Buruli ulcer whenever they visited. Also, 91.82% of the respondents mentioned that they were examined by the volunteers whenever they visited. Majority (91.0%) of the respondents said that they were happy with the work of the volunteers. Interestingly, 92.6% of the respondents indicated that they would like the visit to continue with the majority (67.6%) opting for once in a month visit, just as was done during the project implementation.

## Discussion

This study has demonstrated that it was possible to establish surveillance response system to conduct physical examination on participants on monthly basis. In the context of BU, this may enhance early case detection, diagnosis and treatment. The surveillance-response system implemented was not only able to pick cases at early stages to eliminate or, at least, reduce severe and debilitating ulcers associated with late reporting at health facilities, which causes morbidity and disabilities, but also rediscovered an old ulcerated case that was hidden from the view of the health system. This confirmed the report that patients are not only reporting late at health facilities but many more maybe hidden in the communities [16] and this may require active community-based surveillance to discover and support their diagnosis and treatment.

The surveillance system was able to pick all categories of BU lesions based on WHO (2008) categorization; c*ategory I—*a single lesion <5 cm in diameter; c*ategory II—*a single lesion between 5 and 15 cm in diameter; *category III—*a single lesion >15 cm in diameter, multiple lesions, lesion(s) at critical sites (eye, breast, genitalia) and osteomyelitis [23].

Proper community engagements and training of community-based volunteers, nominated by community members themselves to implement the surveillance-responses system has contributed to the acceptance and participation of the people in the project activities, where on average, each person received at least 11 surveillance contacts with physical examination done on over 90% of them. It was possible to achieve this because efforts were made to follow all community protocols and encouraged the people to see the project as their own. It was reported from a malaria study that, community participation is vital for the success of community based interventions and to achieve this may require full engagement of community members in the process from the start of the project, making them to claim ownership of it by observing existing community protocols and respecting established hierarchy of power within the study community [25].

With reduction in BU cases in endemic communities, other skin conditions must be integrated into BU surveillance-response system that is established to make it more cost effective. In our study, the other skin conditions were referred to the health facilities for management and most of them were treated without any laboratory diagnosis. Though the patients were treated, it will have been better if they were taken through diagnosis to know exactly what conditions were being treated. This may be useful to determine the kinds of lesions circulating in the study communities, to aid the design of any control and prevention strategies.

The socio-demographic characteristics of the study area has remained virtually the same as was reported in earlier studies [12,15,17,18,26], where the Ga ethnic groups were dominant with majority of them identifying with the Christian religion with primary level education. The knowledge of the disease was very high among respondents, gained mostly through the experiences of knowing someone with the infections. It was interesting to note that the socio-demographic characteristics did not affect the level of BU knowledge in the study area, implying that BU-related knowledge is evenly spread among the population. It was encouraging to know that majority of respondents could recognized early suspected lesions and this must be promoted to encourage early reporting at health facilities to promote early diagnosis and treatment as this may help reduce the disease burden on the individual, the affected family and the health system, since the current treatment is very effective when the infection is treated early [21].

Community-based volunteers have been used in Ghana in varied ways to support community-based public health service delivery. However, in the context of BU control, they have been involved in passive case finding in their communities and the added value for using them in this study was their involvement in active case finding through surveillance home visits, which makes it difficult for them to miss any case in the community. The volunteers were motivated with a token, which is about $20 every month to cover their communication and any other incidental cost that they might have incurred during surveillance rounds. In addition, bicycles were given to them to aid mobility during monthly rounds and this has been attested to by respondents as very helpful. The motivation was not solely in the token given to them but also in the fact that they saw themselves as contributing to bringing good health to their people and it was encouraging to hear some respondents asking for the continuation of the project, so that they could be visited at home monthly. This is an indication that with good community entry, respect for local authorities and rapports, it is possible to conduct community-based surveillance with over 90% bodily examinations in BU endemic communities. Since efforts in determining the transmission rout remains elusive, we could take advantage of the acceptance of the community-based surveillance-response system to not only pick cases early but also discover old but hidden ulcers for diagnosis and treatment.

A major limitation of the study was that, it was designed only to confirm BU lesions but not to test for any other cutaneous NTDs and this must be addressed in subsequent studies, given the current downward trend of BU cases in endemic communities including our study area. Secondly, the study period of 12 months was rather short for a longitudinal study of this nature.

The study was setup to determine the feasibility of training periphery health workers and community-based volunteers to implement a community-based active surveillance–response system for early buruli ulcer case detection, diagnosis and treatment at outpatient clinics. At the end of the study, it can be concluded that, it is feasible to train periphery health workers and community-based volunteers to carry out community-based surveillance-response system for early BU case detection, diagnosis and treatment. Given the high number of non-BU skin lesions detected during the 12 month surveillance period, it is recommended that any BU surveillance response system must be an integrated one to aid the detection, diagnosis and treatment of other skin conditions. This will make such an intervention more cost effective as the number of BU cases continues to decline in most endemic communities in Ghana after the introduction of antibiotics treatment.

## Supporting information

S1 BU SURVEILLANCE RECORD FORM (DOCX)

S1 BU CENSUS FORM (DOCX)
