# Supplementary material for: Implementing active community-based surveillance-response system for Buruli ulcer early case detection and management in Ghana
Source: PLoS Negl Trop Dis. 2018 Sep 12;12(9):e0006776. doi: 10.1371/journal.pntd.0006776 (PMC6152995; doi:10.1371/journal.pntd.0006776)
Supplement: S1 BU SURVEILLANCE RECORD FORM — (DOCX) [file pntd.0006776.s001.docx]

#
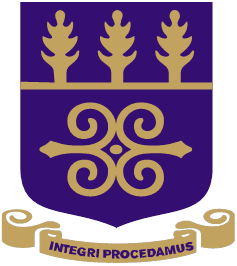


# NOGUCHI MEMORIAL INSTITUTE FOR MEDICAL RESEARCH

# S1_BU SURVEILLANCE RECORD FORM (FORM 2)

| SECTION A: BACKGROUND DETAILS | | | **Response** |
| --- | --- | --- | --- |
| **A1** | Date of Visit (dd/mm/yy | | / / |
| **A2** | A2 Community Zone Number | | # |
| **A3** | Community | | # |
| **A4** | House Number | | # |
| **A5** | Participant’s Serial Number from Census Register | | # |
| **A6** | Participant’s Unique ID (A2+A3+A4+A5) | |  |
| **A7** | Date of Birth (dd/mm/yy) | | / / |
| **A8** | Age (in years) | |  |
| **A9** | Gender | Male | 1 |
|  |  | Female | 2 |
| **A10** | Community Name: | | |
| **A11** | Participant’s name: | | |

| **SECTION B: SURVEILLANCE VISIT AND EXAMINATIONS** | | | | **Response** |
| --- | --- | --- | --- | --- |
| **B1a** | Participant Present at home | **B1a Visit 1** | Yes | 1 |
|  |  |  | No | 2 |
| **B1b** |  | **B1b Visit 2** | Yes | 1 |
|  |  |  | No | 2 |
| **B1c** |  | **B1c Visit 3** | Yes | 1 |
|  |  |  | No | 2 |
| **B2** | Bodily Examination Done on participant | Yes | | 1 |
|  |  | No | | 2 |
| **B2a** | If no, provide reason  (**select ‘not at home’ only after the 3^rd^ visit**) | Refused | | 1 |
|  |  | Not at home | | 2 |
|  |  | Travelled | | 3 |
|  |  | Move out of community | | 4 |
|  |  | Other specify | | 66 |
| **B3** | Suspected Lesion Found  (**If yes administer form 3 in addition to this form**) | Yes | | 1 |
|  |  | No | | 2 |
| **B4** | Location of lesion | Left Arm | | 1 |
|  |  | Right Arm | | 2 |
|  |  | Left Leg | | 3 |
|  |  | Right Leg | | 4 |
|  |  | Head | | 5 |
|  |  | Face | | 6 |
|  |  | Back | | 7 |
|  |  | Stomach | | 8 |
|  |  | Buttock | | 9 |
|  |  | Other Specify | | 66 |

| **SECTION C: EXAMINATION OF OTHER HEALTH CONDITIONS** | | | **Response** |
| --- | --- | --- | --- |
| **Symptoms in the past two weeks including now** | | |  |
| **C1** | Fever | Yes | 1 |
|  |  | No | 2 |
| **C2** | Headache | Yes | 1 |
|  |  | No | 2 |
| **C3** | Vomiting | Yes | 1 |
|  |  | No | 2 |
| **C4** | Chills & Rigors | Yes | 1 |
|  |  | No | 2 |
| **C5** | Convulsions | Yes | 1 |
|  |  | No | 2 |
| **C6** | Diarrhoea | Yes | 1 |
|  |  | No | 2 |
| **C7** | Others (specify) | | 66 |
| **C8** | BP Taken | Yes | 1 |
|  |  | No | 2 |

**BP Classification (Select only one)**

| **C8a** | **Systolic (mmHg)** |  | **Diastolic (mmHg** | **Response** |
| --- | --- | --- | --- | --- |
| Optimal | <120 | and | <80 | 1 |
| Pre-hypertension | 120-139 | and/or | 80-89 | 2 |
| Stage 1 HPT | 140-159 | and/or | 90-99 | 3 |
| Stage 2 HPT | 160-179 | and/or | 100-109 | 4 |
| Stage 3 HPT | ≥180 | and/or | ≥110 | 5 |

**Based on the classification of Rashid and colleagues (2011)**

| **D1** | GIS co-ordinates of Compound | E: | N: |
| --- | --- | --- | --- |

Signature (Community Assistant):________________________ Date (dd/mm/yy): ____/____/____/
