# Supplementary material for: Implementing active community-based surveillance-response system for Buruli ulcer early case detection and management in Ghana
Source: PLoS Negl Trop Dis. 2018 Sep 12;12(9):e0006776. doi: 10.1371/journal.pntd.0006776 (PMC6152995; doi:10.1371/journal.pntd.0006776)
Supplement: S1 BU CENSUS FORM — (DOCX) [file pntd.0006776.s002.docx]

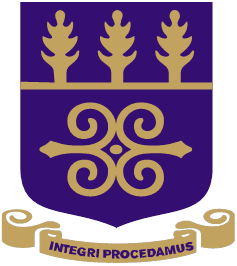


NOGUCHI MEMORIAL INSTITUTE FOR MEDICAL RESEARCH

**S2_HOUSEHOLDS CENSUS AND DEMOGRAPHIC DATA FORM**

**GA WEST MUNICIPALITY, GREATER ACCRA REGION**

Form 1 (NO TWO HOUSEHOLDS ON ONE SHEET)

**PROJECT TITLE:** BURULI ULCER CONTROL IN GHANA: ESTABLISHING ACTIVE COMMUNITY-BASED SURVEILLANCE-RESPONSE SYSTEM FOR EARLY CASE DETECTION AND MANAGEMENT

1. Date: ___ /__ _/___ (*dd/mm/yy*)

2. VILLAGE: ______________________ 3. House Co-ordinates: ____________________

4. House No.: 5. Household code ________________________

6. General Information:

**Head of household or his/her representative to be interviewed**

| 6a. Serial No | **6b.** Name | **6c.** Date of Birth/ Age | **6d.** Sex  M=1  F= 2 | **6e.** Highest level of Education  **(code from list)** | **6f.** Main Occupation  **(code from list)** | **6h. Religion**  **(code from list)** |
| --- | --- | --- | --- | --- | --- | --- |
| 1 |  |  |  |  |  |  |
| 2 |  |  |  |  |  |  |
| 3 |  |  |  |  |  |  |
| 4 |  |  |  |  |  |  |
| 5 |  |  |  |  |  |  |
| 6 |  |  |  |  |  |  |
| 7 |  |  |  |  |  |  |
| 8 |  |  |  |  |  |  |
| 9 |  |  |  |  |  |  |
| 10 |  |  |  |  |  |  |
| 11 |  |  |  |  |  |  |
| 12 |  |  |  |  |  |  |

| **Education** | **Main Occupation** | | **Religion** |
| --- | --- | --- | --- |
| 1=No formal education | 1=Unemployed (18+ years) | | 1=Traditional |
| 2=Primary school | 2=Student/Pupil | | 2=Christian |
| 3=Middle/JHS | 3=Teacher | 7= Sand wining | 3=Islamic |
| 4=Secondary | 4=Fishing | 8=Artisan | 66=Other specify |
| 5=Tertiary | 5=Farming | 9=Unskilled labour |  |
| 6=Non-formal education | 6=Trading | 66=Other specify |  |

**Code from the list provide in the table**

| **6a. Serial No** | **6i. Ethnicity:**  1= Ga/Adangbe  2=Akan  3=Ewe  4=Mole Dagbani  66=Other specify | **Marital Status:**  1=Single  2=Married  3=Divorced  4=Widowed |
| --- | --- | --- |
| 1 |  |  |
| 2 |  |  |
| 3 |  |  |
| 4 |  |  |
| 5 |  |  |
| 4 |  |  |
| 5 |  |  |
| 6 |  |  |
| 7 |  |  |
| 8 |  |  |
| 9 |  |  |
| 10 |  |  |
| 11 |  |  |
| 12 |  |  |

**Code as appropriate from the list bellow based on respondent’s spontaneous response**

| 7 | What is the estimated annual income of your family | GH₵ |  |
| --- | --- | --- | --- |

1= <12,000 2=12,000-20,999

3=21,000-30,999 4=31,000-40,999

5=>41,000

| 8 | Select what describes your House type best | | Cement with zinc/aluminium roofing | | | 1 |
| --- | --- | --- | --- | --- | --- | --- |
|  |  |  | Cement with thatch roofing | | | 2 |
|  |  |  | Mud with zinc/aluminium roofing | | | 3 |
|  |  |  | Mud with thatch roofing | | | 4 |
|  |  |  | Wood with zinc/aluminium roofing | | | 5 |
|  |  |  | Wood with thatch roofing | | | 6 |
|  |  |  | Others; Specify | | | 77 |
|  | | | | | | |
| 9 | How many sleeping spaces/rooms do the household have? | | | |  | |
|  | | | | | | |
| 10 | Does the household have a bed net | | | Yes | | 1 |
|  |  |  |  | No | | 2 |
|  | | | | | | |
| 11 | Any other relevant information |  | | | | |
|  |  |  | | | | |
|  |  |  | | | | |

Name of interviewer: ……………………… Signature of interviewer: …………….
